# Impact of COVID-19 on deaths from respiratory diseases: Panel data evidence from Chile

**DOI:** 10.1080/20008686.2021.2023939

**Published:** 2022-01-28

**Authors:** Claudia Barría-Sandoval, Guillermo Ferreira, Angie Méndez, María Cecilia Toffoletto

**Affiliations:** a Nursing School, Universidad de Las Américas, Concepción, Chile; b Faculty of Nursing, Universidad de Concepción, Concepción, Chile; c Department of Statistics, Universidad de Concepción, Concepción, Chile; d ANID - Millennium Science Initiative Program - Millennium Nucleus Center for the Discovery of Structures in Complex Data, Santiago, Chile; e Nursing School, Universidad de Las Américas, Santiago, Chile

**Keywords:** Respiratory diseases, panel data regression analysis, SARS-CoV-2

## Abstract

The COVID-19 pandemic has relegated pathologies that were previously commonplace to a secondary context. Therefore, it is necessary to study the evolution of these diseases in the presence or absence of COVID-19.

**Objective:**

The present study had the following objectives: 1. to evaluate the relationship between the COVID-19 epidemic and the possible decrease in death from respiratory disease (DRd) in Chile; and 2. to study the relationships between meteorological variables and severity of COVID-19 with respect to DRd from January 2018 to February 2021.

**Methods:**

The variable number of DRds in Chile was analyzed considering the monthly records of meteorological variables (temperature, precipitation and humidity) in each region of Chile and the severity of COVID-19 to evaluate the mortality trend before and after the pandemic. For this, different nonobservable heterogeneity models for panel data were used.

**Results:**

The variables that affect DRd include the number of deaths from COVID-19, which led to a decrease in DRd (negative effect) when increased, the number of patients with COVID-19 in the intensive care unit (ICU), which led to an increase in DRd (positive effect) when increased, and the minimum temperature, which had a negative effect on DRd. These results are supported by the application of panel regression with one-way random-effects models.

**Conclusion:**

This study revealed a reduction in the number of DRds other than COVID-19 during the pandemic in Chile. This could be explained by the sanitary measures applied by the Ministry of Health of Chile in relation to mobility restrictions and social distancing, among others. Therefore, DRd decreased in accordance with the appearance of the COVID-19 pandemic.

## Introduction

In December 2019, the People’s Republic of China reported a cluster of pneumonia cases of unknown etiology, subsequently identified as a new coronavirus by the Chinese Center for Disease Control and Prevention. Later, in February 2020, the World Health Organization (WHO) named the disease COVID-19, short for ‘coronavirus disease 2019’, and in March of the same year, declared an international public health emergency due to its rapid community, regional and global spread. From the confirmation of the first COVID-19 cases to March 2021, 116,736,437 confirmed cases were reported, including 2,593,285 deaths worldwide, of which 45% of confirmed cases and 48% of deaths were within the Region of the Americas. Specifically, South America contributed a higher proportion of deaths (85%) of the total, greatly surpassing North America (14.5%) [1].

In March 2021, the Ministry of Health of Chile (MINSAL, the Spanish acronym for Ministry of Health) published an epidemiological report [2] that registered a total of 1,018,677 cases (confirmed and probable) and a total of 28,756 deaths from COVID-19. Another report [3], also prepared by the MINSAL, describes the analysis of the causes of deaths from COVID-19, where Chile applied rules for coding mortality and simulating the profile of deaths from COVID-19, not considering the existence of the virus as a cause of death in medical certification. This showed that respiratory issues represent the foremost cause of deaths in the country: influenza and pneumonia account for 47%, chronic diseases of the lower respiratory tract account for 3%, respiratory diseases that mainly affect the interstitium account for 3% and other diseases of the respiratory system account for 5%, displacing even hypertensive diseases (4%), cerebrovascular diseases (3%), malignant tumors of the digestive organs (2%), diabetes mellitus (2%), liver diseases (2%), ischemic heart diseases (2%) and others (27%).

In Chile, in 2018 (the prepandemic period), respiratory diseases ranked third in the national ranking of cause of death, surpassed only by diseases of the circulatory system and cancer, with a total of 12,228 deaths from this cause [4]. However, as indicated in the previous paragraph, this situation changed with the arrival of COVID-19 to the country.

A recent study published by [5] revealed that 545 million people currently live with respiratory diseases, which represents 7.4% of the world population, and that 3.9 million people die each year from respiratory diseases, which constitutes a great global health burden due to respiratory disease. The associated healthcare costs are an increasing burden on the economies of all countries, and if the loss of family or caregiver productivity of people with respiratory diseases is considered, the cost to society is much higher [6]. Likewise, at the national level, in people over 65 years of age, respiratory diseases are the second-leading cause of hospital admission [7]. The previous discussion reflects the need to carry out a study on respiratory diseases in the pre- and postpandemic period so that health entities are aware of a quantitative measure of how COVID-19 has affected the statistics on death due to respiratory disease (DRd). This information can then contribute to the effective distribution of health resources.

Several studies have addressed the health impacts relating to mortality from both respiratory diseases and COVID-19. In this context, a Brazilian study made comparisons between DRds and deaths from COVID-19 with a time series statistical methodology for monthly registry data to describe the mortality peak during the summer season. This study revealed a latitudinal gradient in the country, with a peak of 27 deaths occurring in April in most of the northern and northeastern states, and gradually occurring later in the southernmost states, with peak mortality in June and July (cold season) in the Southeast and South regions, respectively [8].

Moreover, a Spanish study used four regression models to explore the associations of the average daily temperature and air quality (PM 2.5) with new daily cases of COVID-19 in the four main regions of Spain (Castilla y Léon, Castilla-La Mancha, Catalonia and Madrid) [9]. Among these models are panel regression models, quantile regression models, clustered OLS models and fixed-effects regression models, known as individual heterogeneity models. Recently, one study used individual heterogeneity models to examine the effects of COVID-19 using the variables of confirmed cases and deaths, which reflect the severity of the pandemic, meteorological factors and air pollutants (PM 2.5), in six countries in South Asia. The authors concluded that high temperature and humidity increase the transmission of COVID-19 and that this can also be applied to regions with higher transmission rates, where the minimum temperature is mostly above 21◦C and the humidity hovers at approximately 80% for months. Additionally, air pollutants (PM 2.5) exhibit significant negative and positive effects on the number of confirmed COVID-19 cases [10]. Other investigations have also used individual heterogeneity models as statistical methodologies to examine the relationships between meteorological variables and environmental pollution with respect to confirmed cases of COVID-19 and mortality, e.g. [11,12], and their references.

Another study evaluated coinfection with COVID-19 in the presence of other respiratory viruses, analyzing the statistically significant correlations between the variables. In particular [13], evaluated the presence of influenza A/B virus, human metapneumovirus, bocavirus, adenovirus, respiratory syncytial virus, and parainfluenza virus in 105 patients who died from COVID-19, finding coinfection with influenza virus in 22.3%, respiratory syncytial virus and bocavirus in 9.7%, parainfluenza virus in 3.9%, human metapneumovirus in 2.9% and adenovirus in 1.9% of COVID-19-positive deaths. Studies have also explored whether COVID-19 has affected the seasonal trends of other viruses; for example, the circulation of influenza has decreased due to the implementation of sanitary measures, such as facial protection, hand hygiene, physical distancing, quarantines, and border closures, among other preventive measures [14,15]. However, it circulates together with COVID-19 and is able to generate greater impacts on the evolution and management of respiratory diseases [16]. In this context [17], expressed that the COVID-19 pandemic impacted influenza epidemiology and surveillance with the consequent undernotification and detection; therefore, they suggest joint surveillance between influenza and COVID-19 to optimize health interventions.

An important aspect that has aroused great interest among different scientific groups is the relationship between influenza and the COVID-19 outbreak. In this context, a study [18] analyzed whether influenza vaccination minimized the spread of COVID-19 among Italians aged 48–64 years and 65 years and older, concluding that influenza vaccination in people aged 65 years and older was associated with reduced spread and less severe clinical presentation of COVID-19. Readers can dive more deeply into this topic by reviewing the studies carried out in Europe and the USA [19,20]: and [21]. In this study, we focus on respiratory diseases, classified as J00-J99 according to the International Classification of Diseases (ICD 10), that constitute pathologies of the respiratory tract and other structures of the lung associated with morbidity and mortality worldwide [22].

In particular, this research proposes to quantify the relationship between DRd and COVID-19 and meteorological variables in Chile. To do this, we generate a data panel with monthly records from different regions of Chile from January 2018 to February 2021, and individual heterogeneity models are proposed, such as fixed- and random-effects regression models. To the best of our knowledge, this is the first study that attempts to examine the effect of the COVID-19 outbreak and meteorological factors on DRd in 14 regions of Chile from January 2017 to February 2021 using regression analysis of panel data that are robust to heterogeneity across regions. The objective of this work is to provide an empirical study to determine the variables that have affected mortality from respiratory diseases in Chile during the COVID-19 pandemic. We hope this will contribute useful statistical information for government authorities regarding the evolution of DRd in Chile with respect to the COVID-19 pandemic and meteorological variables. A highlight from this study is that, once COVID-19 is controlled in the population, chronic diseases that have been neglected will reappear with renewed force. Therefore, having information on the effects of COVID-19 on these chronic diseases is crucial to effectively allocate human and financial resources. In this context, this study has focused on DRd, which can be extended to other chronic diseases that must be continuously monitored by health authorities.

The rest of the article is organized as follows: Section 1 introduces the data and defines the variables, along with the empirical models, Section 2 reports the empirical results, Section 3 presents the strengths and weaknesses of this study through a brief discussion, and the last section concludes the study.

## Materials and Methods

### Data sources and the definition of variables

**Figure 1.**
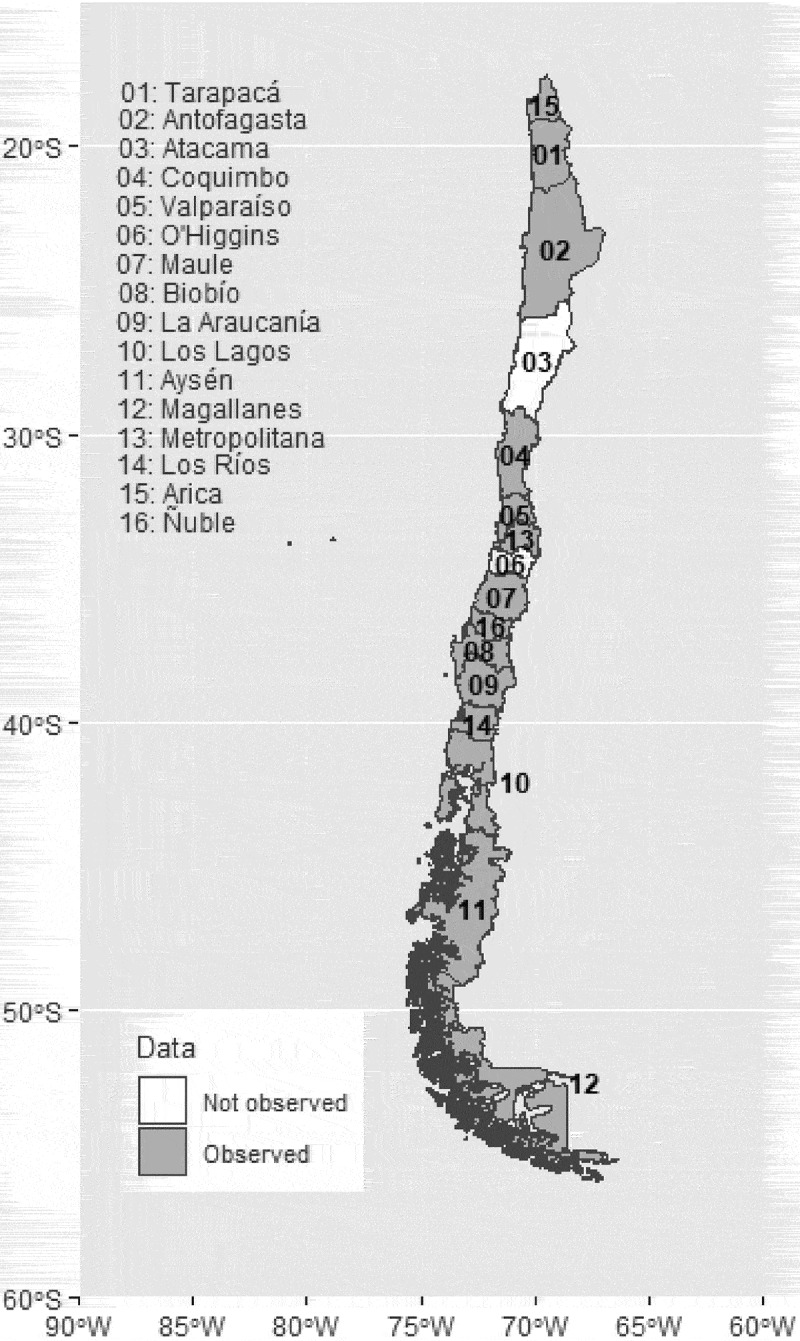
Chile region dataset.

To carry out this study, the monthly records of the variables (Table 1) were considered for 14 regions of Chile from January 2018 to February 2021. These variables include data on the number of DRds obtained from the Ministry of Health https://deis.minsal.cl/, whereas the data regarding the severity of COVID-19 were obtained from the Ministry of Science and Technology https://www.minciencia.gob.cl/covid19. Finally, the temperature and precipitation variable data were obtained from the updated report of the Chilean Meteorological Directorate DGAC, which is available on the website https://climatologia.meteochile.gob.cl. With this information, a balanced data panel was generated with a total of 14 regions and 38 monthly observations. Figure 1 shows a map of Chile with the regions where data recording was possible indicated by gray color. In the regions highlighted in white, it was not possible to build the observation panel since there are no meteorological stations in these geographical areas that provide information on temperature and precipitation. Table 1 describes the variables used in this study. In particular, the dependent variable is the number of DRd. In addition, to investigate the impact of COVID-19 on the number of DRd, we considered three variables: the monthly number of confirmed deaths from COVID-19 (CD), the incidence rate (IC), and the monthly number of patients in the intensive care unit diagnosed with COVID-19 (ICU).

**Table 1.** Variables under study

Type of variable	Variables	Variable code
Explained variable	Death from respiratory disease	DRd
Explanatory variables	COVID-19 death cases	*CD*
	COVID-19 incidence rate	*CI*
	Patients in intensive care unit	I*CU*
	Post & Pre-COVID-19	*Pdummy*
	Maximum temperature	*maxT*
	Minimum temperature	*minT*
	Precipitation	*P*

### Empirical models

To quantify the impact of COVID-19 on the number of DRds in Chile, we used a panel data regression. The panel data models take into account time and regions simultaneously, while other models only express these heterogeneities across units or over time. Furthermore, panel data models are better at capturing the heterogeneity involved in both cross-sectional units and time dimensions to reduce estimation bias and multicollinearity and are better suited for studying the dynamics of change and complex behavioral models [23] and [24]. In this study, we consider two types of panel data models. The first, denoted by Model 1, is a two-way fixed- or random-effects model; that is, this model captures the individual and temporal effects of the data. The second model, called Model 2, is a one-way random- or fixed-effects model, which only considers the temporal effect of the data.

### Two-way fixed-effects model

First, we consider a two-way fixed-effects model with all the explanatory variables as follows

#### Model 1

$\begin{array}{rl} & \log{\left(DRd\right)}_{it}=\alpha +\beta_{1}\log{\left(CD\right)}_{it}+\\ & \beta_{2}CI_{it}+\beta_{3}minT_{it}+\beta_{4}ICU_{it}+\beta_{5}maxT_{it}+\beta_{6}P_{it}+u_{it} \end{array}$

(1) $$u_{it}=\delta_{i}+\eta_{t}+\varepsilon_{it},$$

where DRd denotes the number of deaths from respiratory diseases, CD is the number of monthly deaths from COVID-19, log (CD) is with respect to the observations obtained at the beginning of the pandemic observed from March 2020, CI is the incidence rate, which is the number of monthly confirmed cases divided by the gross permanent resident population (number confirmed/population (10,000 per unit)), *maxT* and *minT* denote temperatures (measured in Celsius), P denotes precipitation (measured in hPa), ICU is the number of COVID-19 patients in intensive care, i denotes the region, t denotes the period under study, $\delta_{i}$ and $\eta_{t}$ are the region and time fixed effects, respectively, and εit denotes the error term. In the case of considering a two-way random-effects model, the individual and temporal effects satisfy the following: $\delta_{i}∼N\left(0,\sigma_{\delta }^{2}\right),\eta_{t}∼N\left(0,\sigma_{\eta }^{2}\right),\varepsilon_{it}∼N\left(0,\sigma_{\varepsilon }^{2}\right)$ and

$$E\left(\delta_{i},\delta_{j}\right)=0,\;\;if\;\;i\neq j,E\left(\eta_{t},\eta_{s}\right)=0,\;\;if\;\;,s\neq t,E\left(\delta_{i},\eta_{t}\right)=0,\;\;\forall i,t.$$

For the random-effects models, there are three estimation methodologies for the parameters, which are available in the free ***R*** statistical software [25], through the *plmtest* command of the *plm* package. This command provides the following methods: *‘swar’* [26] (default), *‘amemiya’* [27], and *‘walhus’* [28].

### One-way fixed-effects model

The data come from 14 different regions, each with peculiar characteristics, which could suggest that the observations from the same region share some common characteristics, such as climatic conditions and economic development. Therefore, such characteristics could be related to the regressors; thus, it is advisable to incorporate the 14 regions as factors in the model and thus control by region. For these reasons, a model is proposed in which the errors $u_{it}$ consider only a time effect to control the contemporary correlation in the cross section, as follows:

#### Model 2

$$\log{\left(DRd\right)}_{it}=\alpha +\beta_{1}\log{\left(CD\right)}_{it}+\beta_{2}CI_{it}+\beta_{3}minT_{it}+\beta_{4}ICU_{it}+\beta_{5}maxT_{it}+\beta_{6}P_{it}+s_{it}+u_{it}$$

(2) $$u_{it}=\eta_{t}+\varepsilon_{it},$$

where $s_{it}=\delta_{i}R_{i}I_{i}\left(t\right)$ with $R_{i}$ represents the i-th region and $I_{i}\left(t\right)\in \left\{0,1\right\}$ an indicator variable, for i= 1,…, 14,t= 1, …,T, defined by

$$I_{i}\left(t\right)=\left\{\begin{array}{c} 1,t=\left(i-1\right)T+1,\ldots ,iT\\ 0,otherwise. \end{array}\right.$$

## Results

To determine the behavior of the DRd variable in all of the regions registered before and after COVID-19, we consider the prior and posterior mean-variance tests, where the data from January 2020 are posterior to the pandemic, and other months are prior to the pandemic. Table 2 reports the results obtained from applying the mean variance tests. We differentiated the cases of nonhomogeneity of variance, in which case we use Welch’s test. From Table 2, we can determine that the number of DRds in all the study regions is significantly lower after than before the outbreak, except in the Aysén region, which exhibits a nonsignificant difference.

**Table 2.** Mean-variance tests. This table reports the results of the mean-variance test of the number of deaths due to respiratory diseases prior and posterior to the pandemic

	Prior to the pandemic			Posterior to the pandemic			t-welch
Regions	obs	mean	variance	obs	mean	variance	mean (post.)-mean (prior)
Tarapacá	27	13.29	24.60	11	10.36	12.25	−2.93**
Antofagasta	27	27.37	40.47	11	20.45	16.47	−6.92***
Coquimbo	27	40.89	147.51	11	29.30	52.68	−11.59***
Valparaíso	27	122.58	1374.09	11	80.17	326.15	−42.41***
Maule	27	68.23	360.90	11	52.42	170.27	−16.81***
Biobío	27	93.80	770.88	11	65.08	224.63	−28.72***
Araucanía	27	71.19	266.08	11	46.75	87.47	−24.44***
Los Lagos	27	49.67	207.69	11	35.36	22.65	−12.30***
Aysén	27	4.12	5.73	11	3.82	2.57	−0.29
Magallanes	27	8.89	11.49	11	5.19	2.76	−3.71***
Metropolitana	27	437.38	21,688.49	11	350.83	3688.88	−86.55***
Los Ríos	27	22.96	61.19	11	15.82	28.56	−7.14***
Arica	27	10.18	11.62	11	7.18	7.16	−3.00***
Ñuble	27	29.33	78.92	11	22.55	52.87	−6.79**

The symbols ***, **, and * represent significance levels of 1%, 5%, and 10%, respectively.

To complement the previous analysis, we consider a one-way model with the post-and prepandemic dummy variables to study the effects of the pandemic on DRd in Chile, that is:

(3) $$\log{\left(DRd\right)}_{it}=\alpha_{0}+\beta_{1}Pdummy_{t}+\beta_{2}minT_{it}+\beta_{3}maxT_{it}+\beta_{4}P_{it}+\delta_{i}+\varepsilon_{it},$$

where $Pdummy_{t}$ is a dummy variable, assuming a value of 1 for records after the COVID-19 pandemic and 0 for the months before the COVID-19 pandemic. This dummy variable can be used to directly compare the differences between DRd before and after COVID-19. The models used to describe the relationship of equation (3) include the pooling model, which groups all the cross sections without considering any form of individual effect. The nonobservable heterogeneity models include the within model, which is also called the fixed-effects model, and the random-effects models defined above. Table 3 shows the estimated results of the aforementioned models.

**Table 3.** The individual effect model

	OLS pooling	Fixed effect within	Random effect swar	walhus	amemiya
*Pdummy*	-0.15310 (0.11084)	-0.34139*** (0.02846)	-0.34101*** (0.02890)	0.34091*** (0.02910)	-0.34122*** (0.02848)
*maxT*	0.10695*** (0.01212)	−0.01822* (0.00883)	-0.01595 (0.00876)	-0.01542 (0.00877)	-0.01714* (0.00874)
*minT*	-0.09122*** (0.01385)	-0.02976* (0.01315)	-0.03257* (0.01302)	-0.03322* (0.01303)	-0.03111* (0.01300)
*P*	0.00115 (0.00099)	0.00013 (0.00037)	0.00019 (0.00037)	0.00020 (0.00037)	0.00016 (0.00037)
R^2^ Adj. R^2^ Num. obs.$\sigma_{\varepsilon }$$\sigma_{\delta }$	0.13782 0.13128 532	0.35443 0.33308 532	0.33965 0.33464 532 0.29464 0.84821	0.33618 0.33114 532 0.45444 1.17115	0.34741 0.34245 532 0.29350 1.23975

***p < 0.001; **p < 0.01; *p < 0.05

Note that in all models, the $Pdummy_{t}$ covariate is statistically significant. In particular, Column 4 shows that the COVID-19 pandemic has significantly reduced the number of DRds by 34.12%. Importantly, in the previous analysis, and as indicated in Equation 3, only the regional fixed effect was controlled in the regression. We next consider panel data regression according to Equations (1)- (2). Here, the models proposed in this study (1 and 2) will be studied to analyze the variables that affect DRd in Chile.

### Using two-way fixed-effects model: Model 1

**Figure 2.**
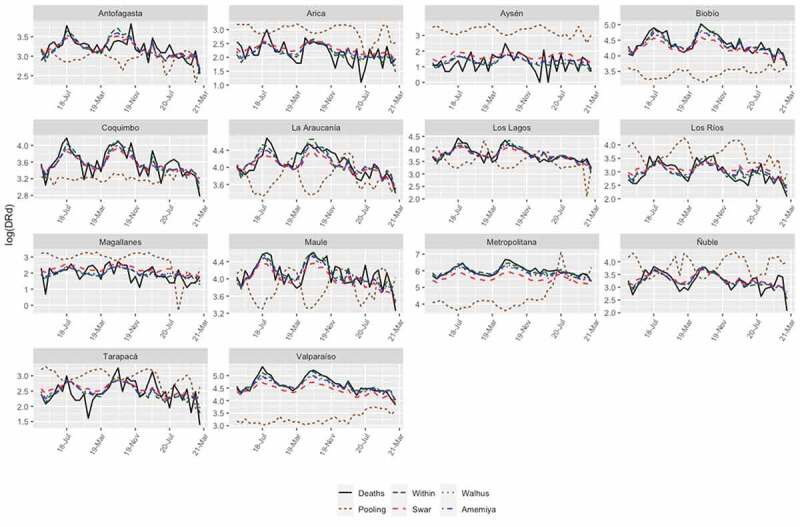
Model 1: Number of DRds other than COVID-19 (red lines) versus fitted values (colored lines).

In this case, the performance of Model 1 was evaluated to determine the degree of impact of the pandemic on DRd in the regions of Chile. For this, we controlled for both the regional fixed effect and the temporal effect in the panel data. Table 4 reports the benchmark estimation results of Model 1. Column 5 shows that the number of deaths from COVID-19 exerted a significant negative effect on the response variable. On the other hand, the number of beds in the ICU used by COVID-19 patients had a significant positive effect. Finally, among the climatic variables, the minimum temperature had a significant negative effect on the number of DRds in Chile, while precipitation had a positive effect. For example, for each percentage unit of increase in deaths from COVID-19, the number of DRds decreased by 7.6%, while for each unit of increase in the number of ICU beds utilized due to COVID-19, the number of DRds increased by 0.055%. Another important aspect is that, if the minimum temperature decreases by one degree, the number of DRds rises by 4.942%, and a unit increase in precipitation would result in an increase of 0.089% in the number of DRds. It is important to note that the performance of Model 1 with respect to adjusted $R^{2}$ is low, which suggests that this model would not be the most appropriate to adjust the data under study. Figure 2 displays the fitted values for each regression model of the data panel, where the black line represents the actual data, and the colored lines represent the fitted values for Model 1. From these figures, we can conclude that the best model to capture the trend structure is a Swar-type random effects model. However, this model does not capture the correlation between individuals. To confirm this, we used Pesaran’s cross-sectional dependence (CD) test [29]. The null hypothesis in Pesaran’s CD test of independence is that residuals across entities are not correlated. The p-value is p < 0.001; therefore, we conclude that there is cross-sectional dependence. As will be analyzed later, Model 2 captures the cross-sectional dependence. In this study, homoscedasticity and serial correlation in idiosyncratic errors were not analyzed since the time dimension of our data was not long enough, as indicated [24].

**Table 4.** The two-way effect model: Estimated parameters of Models 1 on the panel data

	OLS pooling	Fixed effect within	Random effect swar	walhus	amemiya
log (CD)	0.10598* (0.04358)	-0.03090 (0.02171)	-0.05814** (0.01817)	-0.05985*** (0.01760)	-0.07622*** (0.01807)
CI	-0.01308*** (0.00220)	0.00034 (0.00062)	0.00016 (0.00063)	0.00021 (0.00061)	-0.00037 (0.00077)
ICU	0.00255*** (0.00058)	0.00030 (0.00017)	0.00040* (0.00017)	0.00039* (0.00016)	0.00055** (0.00020)
maxT	0.09466*** (0.01153)	0.00543 (0.00877)	0.00438 (0.00847)	0.00185 (0.00836)	0.01278 (0.00906)
minT	-0.08177*** (0.01316)	-0.01078 (0.01489)	-0.02898* (0.01329)	-0.02777* (0.01330)	-0.04942*** (0.01304)
P	0.00071 (0.00093)	0.00071 (0.00039)	0.00078* (0.00038)	0.00073* (0.00037)	0.00089* (0.00043)
$R^{2}$Adj. $R^{2}$ Num. obs.$\sigma_{\varepsilon }$$\sigma_{\delta }$$\sigma_{\eta }$	0.23628 0.22755 532	0.01495 -0.10118 532	0.04244 0.03150 532 0.41075 1.05353 0.36376	0.04704 0.03615 532 0.25793 1.18026 0.22496	0.09284 0.08248 532 0.25955 0.24181 0.10289

***p < 0.001; **p < 0.01; *p < 0.05

### Using one-way fixed-effects model: Model 2

**Figure 3.**
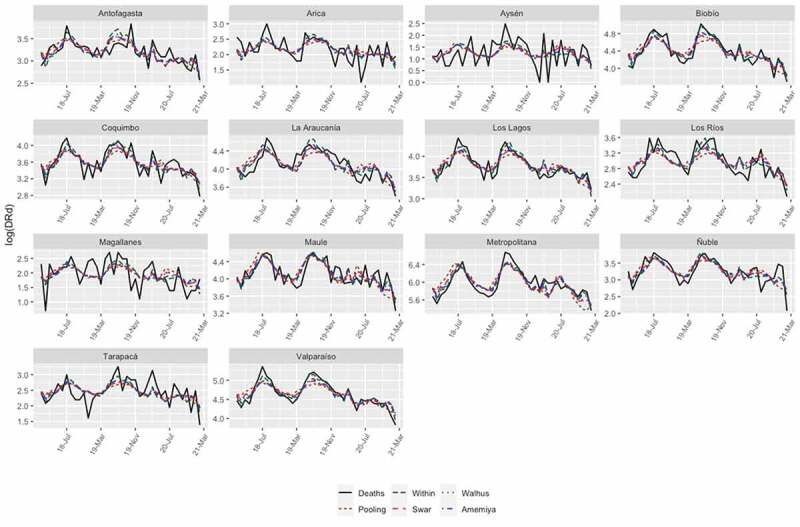
Model 2: Number of DRds other than COVID-19 (black lines) versus fitted values (colored lines).

Chile is one of the most centralized countries in Latin America and of the Organisation for Economic Co-operation and Development (OECD) [30]; that is, there is a high concentration of resources and development in the metropolitan region. Specifically, the capital of the country concentrates the best schools, hospitals, clinics, universities, cultural events and social development, among others. To eliminate the ‘centralization’ effect, the regional effect was used as a covariate to study the impact of COVID-19 on DRd based on the one-way fixed-effects model. Table 5 shows the estimated results with respect to Model 2. From Column 5, it is observed that COVID-19 significantly reduced the number of DRds. For example, a unit percentage increase in deaths from COVID-19 would result in a 5.987% decline in the number of DRds. On the other hand, a unit increase in the number of COVID-19 patients in intensive care leads to a 0.039% increase in the number of DRds. Another important aspect is that, if the minimum temperature decreases by one degree, the number of DRds increases by 2.681%. In this study, the precipitation variable did not explain the number of DRds. This coincides with major regional climate trends, including decreasing precipitation and increasing temperatures, which have caused a megadrought in recent decades in Chile [31]. Unlike Model 1, the performance of Model 2 with respect to the adjusted $R^{2}$ is high; this is supported by Figure 3, which displays the adjusted values of the panel regression model defined in Table 5. This suggests that Model 2 would be the most suitable for fitting the data in the study. A Hausman-type test will determine whether individual effects should be treated as fixed or can be assumed as incorrect with regressors when using a more efficient random-effects specification. Under the hypothesis of no correlation between the regressors and individual effects, this hypothesis, with a p-value of 18%, is not rejected at the 5% confidence level.

**Table 5.** Time effect model: estimated parameters of Models 2 on the panel data

	OLS pooling	Fixed effect within	Random effect swar	walhus	amemiya
log (CD)	-0.10631*** (0.01236)	-0.03090 (0.02171)	-0.08698*** (0.01446)	-0.08258*** (0.01498)	-0.05987*** (0.01763)
ICU	0.00041* (0.00017)	0.00030 (0.00017)	0.00044** (0.00016)	0.00044** (0.00016)	0.00039* (0.00016)
CI	0.00058 (0.00063)	0.00034 (0.00062)	0.00024 (0.00062)	0.00022 (0.00062)	0.00024 (0.00061)
maxT	-0.01190 (0.00884)	0.00543 (0.00877)	-0.00432 (0.00851)	-0.00329 (0.00848)	0.00078 (0.00841)
minT	-0.03934** (0.01319)	-0.01078 (0.01489)	-0.03766** (0.01302)	-0.03627** (0.01307)	-0.02681* (0.01349)
P	0.00015 (0.00037)	0.00071 (0.00039)	0.00057 (0.00037)	0.00061 (0.00037)	0.00071 (0.00037)
$R_{2}$	0.67193*** (0.06807)	0.74910*** (0.06388)	0.68753*** (0.06297)	0.69156*** (0.06251)	0.71412*** (0.06145)
$R_{4}$	0.86748*** (0.08350)	1.08874*** (0.09425)	0.89904*** (0.08108)	0.91035*** (0.08134)	0.97911*** (0.08442)
$R_{5}$	1.98748*** (0.08085)	2.15055*** (0.09018)	2.00265*** (0.07806)	2.01058*** (0.07824)	2.06254*** (0.08088)
$R_{7}$	1.37512*** (0.13627)	1.55945*** (0.14254)	1.36445*** (0.13147)	1.37265*** (0.13133)	1.43866*** (0.13270)
$R_{8}$	1.59892*** (0.11120)	1.83280*** (0.13025)	1.60548*** (0.10968)	1.61631*** (0.11027)	1.69452*** (0.11544)
$R_{9}$	1.20809*** (0.15198)	1.50935*** (0.17435)	1.21023*** (0.14989)	1.22408*** (0.15053)	1.32677*** (0.15638)
$R_{10}$	0.83412*** (0.14260)	1.16997*** (0.17185)	0.84743*** (0.14230)	0.86315*** (0.14334)	0.97483*** (0.15121)
$R_{11}$	-1.85950*** (0.13457)	-1.35747*** (0.17973)	-1.79909*** (0.13828)	-1.77391*** (0.14027)	-1.61523*** (0.15293)
$R_{12}$	-1.17616*** (0.11768)	-0.60818*** (0.17965)	-1.07321*** (0.12574)	-1.04381*** (0.12870)	-0.87372*** (0.14629)
$R_{13}$	3.26685*** (0.15091)	3.39136*** (0.15192)	3.22930*** (0.14459)	3.23398*** (0.14415)	3.28607*** (0.14396)
$R_{14}$	-0.01362 (0.15582)	0.33558 (0.18344)	-0.00656 (0.15495)	0.00963 (0.15588)	0.12778 (0.16320)
$R_{15}$	-0.26138*** (0.06761)	-0.28527*** (0.06022)	-0.26524*** (0.06196)	-0.26645*** (0.06136)	-0.27364*** (0.05942)
$R_{16}$	0.41472** (0.15050)	0.66458*** (0.16328)	0.40898** (0.14658)	0.42034** (0.14674)	0.50774*** (0.14988)
$R^{2}$Adj. $R^{2}$ Num. obs.$\sigma_{\varepsilon }$$\sigma_{\eta }$	0.94595 0.94395 532	0.95901 0.95417 532	0.95330 0.95157 532 0.25955 0.10289	0.95411 0.95241 532 0.26790 0.12376	0.95681 0.95521 532 0.25451 0.22524

***p < 0.001; **p < 0.01; *p < 0.05

Furthermore, Pesaran’s CD test for cross-sectional dependence is not significant with a 5% confidence level (p-value 36%); that is, there is no cross-dependence between individuals. For model selection among the Swar-type model and Amemiya random-effects model, we used the Akaike information criterion (AIC) and Bayesian information criterion (BIC). Table 6 reports the AIC and BIC statistics of Models 1–2. All indicators favor the Amemiya random-effects model, i.e., Model 2. Therefore, a random-effects model with the Amemiya method is more appropriate for our panel data, with an adjusted $R^{2}=0.95521$. Finally, we can observe that the regions of the extreme south (Regions 11 and 12) and extreme north (Regions 1 and 15) show a decrease in the number of DRds compared to the regions of the central zone of the country (Regions 5, 7, 8 and 13). Possible causes for this are the lower demographic density in the extreme regions of the country, limited access to health care services, and the scarce integration of indigenous peoples in public health programs, among others.

**Table 6.** The descriptive statistics of ex post forecast errors and summary of model selection criteria

Model	ME	RMSE	MAE	MPE	MAPE	AIC	BIC
Swar-Model 1	9.39	57.28	29.34	−302.16	337.01	361.22	391.16
Amemiya-Model 2	-22.84	52.37	23.24	-87.31	89.22	84.16	169.70

### Analysis of ex post forecast accuracy

In this section, we evaluate the accuracy of the predictions by using a training set and test set. We consider a training set $X_{it}=DRd_{it}$ from January 2018 to February 2021 (estimation period) with a total of N=38 monthly observations and test data from March 2021 to September 2021 (validation period), which will be used for m-step-ahead prediction with m=7 monthly observations. The values ${\hat{X}}_{i,N+1},\ldots ,{\hat{X}}_{i,N+m}$ are called the ex post forecast or period forecast with the starting period on March 2021. The m-step-ahead forecasts are compared with the validation period, giving rise to ex post forecast errors, i.e., $X_{i,N+h}-{\hat{X}}_{i,N+h}$ for horizon h=1,…,m.

**Figure 4.**
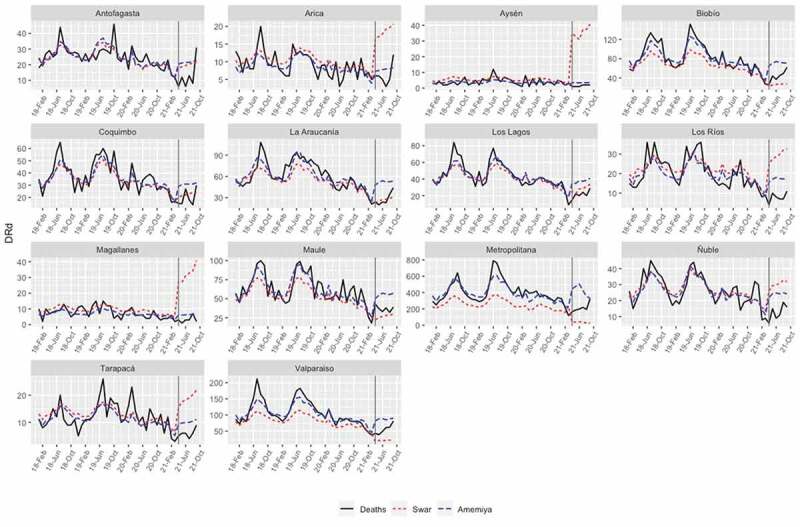
Multistep forecasts for number of DRds other than COVID-19 (black lines).

The errors were assessed by the statistics of the residuals, such as the mean error (ME), root mean square error (RMSE), mean absolute error (MAE), mean percentage error (MPE) and mean absolute percentage error (MAPE), where small values of these statistics reflect the goodness of fit of the model used. Table 6 reports the statistics of ex post forecast errors of the models on both datasets. All indicators favor the Amemiya model, i.e., Model 2. Figure 4 displays the out-of-sample predictions for the 14 regions under study. The Amemiya-Model 2 predictions are closer to the true values than their Swar-Model 1 counterparts, demonstrating the suitability of the proposed model for prediction.

The solid and broken lines represent real values and the ex post and ex ante forecasts, respectively.

## Discussion

An important strength of this study is the general design used to assess the possible relationships of the COVID-19 pandemic with climatic conditions and mortality from respiratory diseases. In all statistical analyses performed in this study, the magnitude and direction of the observed phenomena were found to be consistent. Importantly, by incorporating geographic location in Model 2 as an explanatory variable, the magnitude and statistical significance of the observed results increased considerably.

Regarding the behavior of the number of DRds as a variable in all the regions registered after the pandemic, it was significantly lower than the records prior to the COVID-19 pandemic. These findings confirm the hypothesis of a study conducted in Brazil, in which the authors estimated an average underreporting of 40.68% (range 25.9% −62.7%) for deaths related to COVID-19 in the country [8]. Another possible cause of the decrease in the number of DRds could be related to the health measures implemented in Chile, in which a state of emergency was declared on 18 March 2020, and the implementation of an overnight curfew, nationwide lockdown and regional quarantines until September 2021, measures that were effective in reducing the rate of infections by COVID-19. Currently, the recommendations for social distancing, mandatory use of face masks, and indication of voluntary vaccination continue [16].

In this study, it was also observed (Figure 3) that, in the extreme northern and southern regions of Chile, Amemiya-Model 2 exhibited low performance in explaining the number of DRds. One possible cause is that the population density of these regions is lower than that of other areas. Another possible cause is the large difference in the growth domestic product (GDP) per capita (GDP/Population) between the extreme regions of the country and the metropolitan region [30], which is associated with compliance with confinement measures and the preventive health care available to the inhabitants of these regions.

The findings of the present study have several limitations. First, this study employed a longitudinal design. Therefore, in these types of studies, variables recorded both in time and by locality are needed, and these are difficult to obtain. In this context, the monthly records of several sociodemographic variables were not available by region; therefore, they were not used to analyze the association between COVID-19 and social determinants of health (SDHs), such as race/ethnicity, gender, socioeconomic status, rural/urban residence, and housing status, with respect to avoiding DRd. A subsequent study that not only considers the geographical differences that affect self-protection habits against respiratory diseases and COVID-19 but also incorporates SDHs is proposed.

Second, our findings could have been affected by the number of individuals vaccinated against influenza during the COVID-19 pandemic. Another possible limitation of this study could be the existence of errors in the identification/recording of the true cause of death in the database and errors in the recording/calculation of the climatic conditions analyzed in the database. It is presumed that, if such inaccuracies occurred in the data, they would have occurred with similar frequency among the different databases examined. Therefore, if such phenomena were present in the data examined, the statistical power of this study would have been reduced. Nevertheless, the present study’s results provide some insight into the association between the COVID-19 pandemic and the number of DRds.

## Conclusion

Our findings show that the variables that affect the mortality rate from respiratory diseases include the number of deaths from COVID-19, which has a negative effect; the number of patients with COVID-19 in the ICU, which has a positive effect; and the minimum temperature, which has a negative effect. These results are supported by the application of panel regression for a one-way random-effects model and using the procedure of Amemiya for estimating model parameters. We can conclude that the number of DRds (other than COVID-19) in Chile decreased with the appearance of the COVID-19 pandemic.
